# Mental Health First Aid Training with the Nepalese Community in Australia: An Evaluation of Effects on Knowledge, Confidence, Intentions, Willingness to have Contact and Stigmatizing Attitudes

**DOI:** 10.1007/s10903-022-01397-7

**Published:** 2022-09-08

**Authors:** Bharat Nepal, Gaurav Khadka, Anthony F Jorm, Jyoti Simkhada, Nirajan Gauli, Neil Hall

**Affiliations:** 1 Australia Nepal Public Link Inc, Sydney, Australia; 2 Melbourne School of Population and Global Health, University of Melbourne, Melbourne, Australia; 3 School of Social Sciences, Western Sydney University, Sydney, Australia

**Keywords:** Nepalese, Mental Health First Aid, Stigma

## Abstract

The aim of this study was to evaluate the effectiveness of Mental Health First Aid (MHFA) training amongst the Nepalese community in Australia by exploring the impact on knowledge about mental health first aid, confidence and intentions to help, willingness to have contact and stigmatizing attitudes towards people with mental illness. We hypothesized that since MHFA has been extensively evaluated with other communities and has been found to be effective, it would therefore be effective with this community as well. MHFA training was conducted by an accredited Nepalese-Australian MHFA Instructor with 162 participants from the Nepalese community in four states of Australia. Participants completed an evaluation questionnaire prior to the training (pre-test) and at the end of the training (post-test). The evaluation questionnaire assessed participants? knowledge about what was taught in the course, ability to recognize depression as described in a vignette, confidence in providing help, intentions to provide help, and willingness to have contact and stigmatizing attitudes towards people with mental illness. There were large improvements from pre-test to post-test in knowledge, confidence and intentions to help, medium improvements in willingness to have contact, small-to-medium improvements in stigmatizing attitudes and small improvements in recognition of depression. Participants gave high ratings of the course and the instructor. MHFA training produced improvements in knowledge, confidence, intentions, willingness to have contact and stigmatizing attitudes. The training was also well received. Further research is needed to assess persistence of these effects following the course and any changes in mental health first aid provided to the community.

## Background

The Mental Health First Aid (MHFA) course was developed in Australia in the year 2000 to provide community members with the knowledge and skills to support a person who is developing a mental health problem or in a mental health crisis [1]. A systematic review of randomized controlled trials found that there was strong evidence for improvements in participants’ knowledge about mental health problems at post training, which persisted up to a year afterwards [2]. It also demonstrated moderate improvements in beliefs about appropriate treatments, and the ability to identify a person with a mental health problem, which were sustained up to six months after the training. This review also found that MHFA training led to small reduction in stigmatising attitudes, and moderate to large improvements in intentions to provide first aid to a person with mental health problem. However, there is limited evidence on the effects of MHFA training of ethnic minority populations. The present paper focuses on the potential benefits of MHFA with the Nepalese community in Australia.

The Nepalese community is one of the fastest growing communities in Australia. As of 30 June 2020, the Nepal-born population residing in Australia was the 11th highest amongst overseas born people, representing 1.6 per cent of the Australian population [3]. The growth of the Nepalese population in Australia from 2009 to 2019 has been 400%, with the median age of Nepalese migrants being 27.3 years and almost 55 per cent are male. Australia is one of the most preferred educational destinations for Nepalese students and Nepal is the third largest source of international students in Australia accounting for 6 per cent of all international students [4].

Mental health issues are commonly experienced around the world; one in five adults experience a mental health problem in any year in Australia [5]. A pilot study conducted by the Nepal Health Research Council found a similar prevalence of mental illness in the Nepalese adult population [6]. With the rapid increase of Nepalese permanent and temporary migrants in Australia, there is clearly an increased number of Nepalese community members experiencing mental health issues and requiring professional help. There are several risk factors for poor mental health in the Nepalese community, such as being in a new country and experiencing settlement issues, lack of friends and support network, financial stress, education pressures and unemployment [7]. However, systematic research about the experience of mental health issues in the Nepalese community is severely limited.

Timely and appropriate health-related help seeking is paramount for the improving population mental health and reducing health inequity. A study undertaken in the UK found that the Nepalese population would not access services due to a social taboo around mental health and wellbeing [8]. Researchers [9] have noted that Nepalese community members do not tend to open up and share their issues if these involve taboos. In addition to stigma about mental illness, poor mental health literacy and availability and effectiveness of professional help contribute to the poor uptake of professional help seeking amongst Nepalese community members [10].

### Mental Health Literacy in Nepali Speaking Community in Australia/Internationally

Because mental illness is common in the general community around the world, it is important that members of the public have at least a basic knowledge about common mental health problems and skills needed to help those experiencing mental health problems among their friends or family members. Lack of accurate information about mental health issues acts as a barrier and inhibits appropriate and timely help seeking [11], which results in delay in their recovery.

A pilot study undertaken by Nepal Health Research Council found the prevalence of mental illness amongst Nepalese adults was similar to Australia [6, 12, 13]. However, negative attitudes of community members as well as health professionals towards people with mental illness in Nepal is documented in the literature [14]. Researchers have found that Nepalese community members both in Nepal and in Australia have much higher stigmatizing attitudes compared to the general Australian population [15, 16] in addition to facing challenges of being in a new country. Sociocultural differences and having come from completely different health care settings often create additional barriers in accessing mental health services or support. A nationally representative systematic study of mental health help seeking behaviours of the Nepali speaking community in Nepal found low utilization of mental health services [17].

Cultural isolation is associated with increased levels of psychological distress, while low socio-economic status is correlated with mental illness [18]. Australian Nepalese fall into this category, indicating challenges faced by this growing community in Australia. The Nepalese community residing in the United Kingdom have been found to be underutilising mental health services, which could be attributed to poor mental health literacy. Health service utilisation is important from the service providers’ perspective and often influenced by health beliefs and associated factors [8]. The Nepalese community in Australia may have similar experiences, being migrants in a new country with vastly different health service provision than that of the country of origin.

### Mental Health First Aid

MHFA is widely used to train community members across the world. Within Australia, nearly 3% of Australian adults have received this training since its inception in early 2000.

There is evidence to show that MHFA is culturally acceptable and effective with a number of multicultural communities in Australia and abroad [2];[9];[10];[20]; [25]. Previous evaluation studies with members of the Chinese and the Vietnamese communities in Melbourne Australia found improvements in knowledge and attitudes towards people with mental illness (20; 25). Participants from the Chinese community members demonstrated significant improvement in mental health literacy and reduction in stigmatizing attitudes, while evaluation of MHFA effectiveness in the Vietnamese community demonstrated significant reduction in stigmatizing attitudes and improvement in mental health literacy amongst the participants. Both studies had native speaking instructors, with resources translated and culturally adapted by the mental health professional who delivered the training. In contrast, MHFA training for the Bhutanese refugee community in the USA was delivered by non-native instructors and the resources and evaluation questionnaires were in English language. It was found that the attitude of attaching stigma to mental health issues did not reduce considerably at a post-test evaluation [9], showing that attention to cultural adaptation is needed when the course is used with different cultural groups. It is therefore important to evaluate this intervention when it is used with the Nepalese community in Australia and other countries.

MHFA has been implemented with the Nepalese community in Australia since 2016 and close to 400 participants have been trained until the end of June 2021. However, a formal evaluation of this training has not previously been carried out. Thus, the main aim of this study was to examine the effects of MHFA training on course attendees’ mental health first aid intentions, knowledge about mental health problems and suicide, confidence in providing help and attitudes to people with mental health problems. The post-training evaluation questionnaire also asked about satisfaction with the course and areas for improvement.

## Methods

### Research Design

The course was evaluated in an uncontrolled trial with outcomes measured pre- and post-course.

### Participants

Participants were recruited to the training from Nepalese community members in four States and Territories of Australia (New South Wales, Victoria, Tasmania and Australian Capital Territory) by promoting in Facebook and sending emails to community leaders and community organizations working with the Nepalese community. Selection criteria were being aged 18 years or over and wanting to learn about mental illness and how to provide support to those who are experiencing mental health issues. There was no selection based on how long the person had been in Australia. One hundred and sixty-two participants completed the consent form and the pre/post course questionnaires.

### Training Course

Evaluation of the MHFA training course for Nepalese community members in Australia took place during April 2019 to June 2021. There were 14 Standard 4th edition^1^ MHFA courses delivered to 186 participants, 162 of whom completed pre- and post-training questionnaires. The duration of this course was 12 h over two days, however due to discussion and further explanation in the Nepali language, the course was often extended to 14 h on average.

The MHFA training was primarily organized by Australia Nepal Public Link (ANPL) Incorporated, a not-for-profit community organization registered with the New South Wales Department of Fair Trading and the Department of Community Services in Victoria, Australia. ANPL has been organizing MHFA training for the Nepalese community in Australia free of charge to the participants. The trainer is a native Nepali speaker who has been delivering MHFA for the Nepalese community since 2016. During the course, most of the discussion was carried out in the Nepalese language, as participants often found it easier to ask questions in their native language. However, the resources and training PowerPoint slides and MHFA Manual were in English.

Participants learned about an overview of mental health issues, and how to assist people affected by depression, anxiety disorders including panic attack, psychosis and substance use disorder, as well as suicidal thoughts and non-suicidal self-injury. First aid actions were taught to participants using an action plan known as “ALGEE” to help people who may be experiencing the worsening of their mental health, or who may already be in crisis: (1) **A**pproach the person, assess and assist with any crisis, (2) **L**isten and communicate non-judgmentally, (3) **G**ive support and information, (4) **E**ncourage the person to get appropriate professional help and (5) **E**ncourage other supports [19]. The other supports included self-help and use of online resources.

### Evaluation Questionnaire

An evaluation questionnaire was administered pre- and post-course. The pre-course and post-course questionnaires were identical except that only the pre-course questionnaire asked about sociodemographic characteristics of the participants, while only the post-course questionnaire asked for ratings of the course. Below are descriptions of each of variables measured:

#### Sociodemographic Characteristics

The sociodemographic section of the pre-course questionnaire asked about age group, gender, whether currently living with partner, primary language, country of birth, number of years lived in Australia, whether currently studying, type of student, whether currently working and whether done previous mental health training.

#### Recognition of Depression

Participants were given the following vignette about a fictional person called ‘Jagadish’: “Jagadish is 30 years old. He has been feeling unusually sad and miserable for the last few weeks. Even though he is tired all the time, he has trouble sleeping nearly every night. Jagadish doesn’t feel like eating and has lost weight. He can’t keep his mind on his work and puts off making any decisions. Even day-to-day tasks seem too much for him. This has come to the attention of Jagadish’s boss who is concerned about his lowered productivity. Jagadish feels he will never be happy again and believes his family would be better off without him. Jagadish has been so desperate, he has been thinking of ways to end his life.” Following the vignette, participants were asked an open-ended question: “What, if anything, do you think is wrong with Jagadish? If you think there is nothing wrong with Jagadish please type ‘nothing’”.

Responses to the question about what is wrong with the person in the vignette were blind scored by a research assistant. One of the researchers (Jorm) used a random number generator to mix the pre- and post-course responses for scoring. Responses were scored as correct if they made any mention of “depression” or a synonym (“depressed”, “depressive”). Previous research with a very similar vignette has shown that Australian mental health professionals regard the person as depressed [20]. Recognition of the problem in a vignette has been used in previous evaluations of MHFA training.

#### Intentions to Provide Mental Health First Aid

To assess intentions to provide mental health first aid, participants were asked the following open-ended question: “Imagine Jagadish is someone you have known for a long time and care about. You want to help him. What would you do?”

Responses to this question were blind scored for quality of response. One of the researchers (Jorm) used a random number generator to mix the pre- and post-course responses, which were then scored by a blinded research assistant for adherence to the MHFA Action Plan taught in the course. Scores varied from 0 to 12. This method of scoring has been validated as a predictor of future quality of actions and used in previous evaluations of Mental Health First Aid training.

#### Confidence in Providing Mental Health First Aid

Participants were asked “How confident would you be in your ability to help Jagadish?” and provided with the following options: Very confident, Fairly confident, Slightly confident, Not confident at all, Don’t know. The ratings were scored on a scale from 1 (Not confident at all) to 4 (Very confident). Confidence has been shown to predict subsequent helping behaviour and has been used in previous evaluations of MHFA training (2).

#### Stigmatizing Attitudes

Participants were asked 7 questions about personal stigma towards Jagadish. These were: “Jagadish could make himself better if he wanted; Jagadish’s problem is a sign of personal weakness; Jagadish’s problem is not a real medical illness; Jagadish is dangerous; It is best to avoid Jagadish so that you don’t develop this problem yourself; Jagadish’s problem makes him unpredictable; You would not tell anyone if you had a problem like Jagadish’s”. Each question was rated on a 5-point scale from Strongly Agree to Strongly Disagree, with higher scores indicating greater stigma. These questions were based on the Personal Stigma Scale. Factor analysis of these items in other samples has found components reflecting Weak-not-Sick and Dangerous/Unpredictable attitudes [20]. However, because the factor structure of these stigma items may vary across cultures, they were analysed as single items in the present study.

##### Willingness to Have Contact

Willingness to have contact was measured by a social distance scale. Participants were asked: “Please indicate how willing you would be to: “Move next door to Jagadish; Spend an evening socialising with Jagadish; Make friends with Jagadish; Have Jagadish start working closely with you on a job; Have Jagadish marry into your family”. Items were rated on the following scale: Definitely willing, Probably willing, Probably unwilling, Definitely unwilling, Don’t know. Items were scored from 1 to 4 and summed, with pro-rating used for up to one missing response, giving scores that could range from 5 to 20. Social distance scales have been extensively used as indicators stigma, including in the evaluation of MHFA training.

#### Mental Health First Aid Knowledge

A mental health first aid knowledge questionnaire was used to assess what was taught in the course. This consisted of 18 true/false questions. Answers were scored 1/0 and totaled to give a score out of 18, with pro-rating used for up to 2 missing responses. Similar knowledge scores have been used in previous evaluations of MHFA training [20].

#### Ratings of the Course

The following questions about quality of the course were asked only post-course, with each rated on a 5-point scale: “How relevant was the course content to you?” (from 1 irrelevant to 5 relevant); “Was it easy to ask the instructor questions?” (from 1 very hard to 5 very easy); How would you rate your instructor’s knowledge of the material? (from 1 poor to 5 excellent); “How new was the information in the program to you?” (from 1 nothing new to 5 completely new); “How would you rate your enjoyment of the program?” (from 1 not at all enjoyed to 5 completely enjoyed). Finally, participants were asked an open-ended question: “Is there anything about the course that you think should be changed?”

### Statistical Methods

Scale means at pre- and post-course were compared with paired-samples t-tests. Effect sizes were measured using Cohen’s d (standardized mean difference) with Hedges’ correction. Following Cohen, d values of 0.2 were regarded as small, 0.5 as medium and 0.8 as large [21].

For the dichotomous outcome of recognition of depression, percentages correct pre- and post-course were compared with a McNemar test. The effect size was measured with an odds ratio (OR). Following Chen et al. [22], ORs of 1.68, 3.47, and 6.71 were regarded as small, medium and large, respectively.

The P < 0.05 level was used for statistical significance. Analyses were carried out using IBM SPSS Statistics 27.

#### Ethics Approval

Human Research Ethics approval was obtained from the Human Research Ethics Committee at University of Melbourne, Victoria Australia (Approval No. 1,953,547, 18th March 2019).

## Results

Characteristics of the participants are shown in the Table 1. The majority were female (60.5%), spoke Nepalese at home (98.1%), were born in Nepal (96.3%), and had been in Australia for less than 5 years. Most of the participants (85.8%) had not had prior mental health training.

**Table 1:** Characteristics of the participants

Characteristic	N (%)
Age group	
18–24 years	22 (13.6)
25–34 years	108 (66.7)
35–44 years	20 (12.3)
45–54 years	8 (4.9)
55–64 years	2 (1.2)
65–74 years	2 (1.2)
Gender	
Female	98 (60.5)
Male	64 (39.5)
Currently living with partner	
Yes	108 (66.7)
No	54 (33.3)
Primary language	
English	1 (0.6)
Nepali	159 (98.1)
Other	2 (1.2
Country of birth	
Nepal	156 (96.3)
Other	4 (3.7)
Years lived in Australia	
2	44 (27.2)
2–4	52 (32.1)
5–6	20 (12.3)
7–8	5 (3.1)
9–10	4 (2.5)
>10	36 (22.2)
Other	1 (0.6)
Currently studying	N (%)
Yes	71 (44.1)
No	90 (55.6)
Missing	1 (0.6)
Type of student	
International	77 (47.5)
Local	12 (7.4)
Not a student	69 (42.6)
Missing	4 (2.5)
Currently working	
Yes	121 (74.7)
No	40 (24.7)
Missing	1 (0.6)
Previous mental health training	
Yes	20 (12.3)
No	139 (85.8)
Missing	3 (1.9)

Key findings are presented below in Table 2. The key findings are that there were large improvements in knowledge, confidence and intentions to help, medium improvements in willingness to have contact and the stigma about mental illness being the sign of weakness, and there were small improvements in recognition of depression and the stigma items on ‘not a real illness’, ‘dangerous’ and ‘would not tell anyone’. All of the above findings are statistically significant.

**Table 2:** Comparison of scores on outcome measures pre- and post-course

Outcome measure	N	Pre Mean (SD)	Post Mean (SD)	P-value	Cohen’s d
Knowledge	147	9.81 (1.88)	12.29 (2.45)	< 0.001	0.93
Confidence to help	154	2.88 (0.81)	3.58 (0.60)	< 0.001	0.83
Intentions to help	162	2.85 (1.33)	5.02 (1.77)	< 0.001	1.11
Stigma—make himself better	159	4.18 (0.99)	4.00 (1.22)	0.062	-0.15
Stigma—sign of weakness	157	2.83 (1.32)	2.11 (1.28)	< 0.001	-0.59
Stigma—not real illness	156	2.35 (1.20)	1.98 (1.28)	0.002	-0.25
Stigma– dangerous	158	2.19 (1.15)	1.93 (1.11)	0.003	-0.24
Stigma—best to avoid	159	1.29 (0.71)	1.31 (0.66)	0.784	0.02
Stigma– unpredictable	159	3.42 (1.08)	3.23 (1.30)	0.077	-0.14
Stigma—would not tell anyone	161	1.78 (0.95)	1.53 (0.73)	0.003	-0.24
Social distance^2^	129	7.88 (2.45)	6.70 (2.02)	< 0.001	-0.70
**Outcome measure**	**N**	**Pre %**	**Post %**	**P-value**	**Odds ratio**
Correct recognition of depression	161	46.0	56.5	0.037	1.81

^2^Willingness to have contact with person who has mental illness was measured by a social distance scale

Table 3 shows participants’ ratings of the course, with all of the questions rated above 4 out of 5. The key findings are that participants’ rated high satisfaction about the course material and relevance, content expertise of the instructor, opportunity to ask questions, and interactivity and enjoyment, which may reflect the language of delivery as the course was delivered in a combination of English and Nepali.

**Table 3:** Participant ratings of the course (1-5 scales)

Question	N	Mean (SD)
How relevant was the course content to you?	160	4.82 (0.69)
Was it easy to ask the instructor questions?	160	4.91 (0.40)
How would you rate your instructor’s knowledge of the material?	160	4.93 (0.38)
How new was the information in the program to you?	160	4.01 (0.90)
How would you rate your enjoyment of the program?	160	4.84 (0.39)

## Discussion

The aim of this study was to evaluate the impact of MHFA training on Nepalese community members by measuring changes in knowledge about common mental illness, changes in stigmatizing attitudes towards a person experiencing mental illness and willingness to provide assistance to the person experiencing mental illness. Using Cohen’s [23] definitions of small, medium and large effect sizes, our study found that there were large improvements in knowledge, confidence and intention to help, medium improvements in willingness to have contact (measured by a social distance scale) and the belief that mental illness is a sign of weakness, and small improvements in recognition of depression and beliefs that depression is not a real illness, that a depressed person is dangerous and that the participant would not tell anyone if they had this problem. These effects can be compared to the findings from a meta-analysis of randomized controlled trials of MHFA training from a range of countries [2], which found medium-to-large effects at post-test on knowledge, confidence and intention to help, small-to-medium effects on willingness to have contact, and s small effect on recognition and a very small effect on stigmatizing attitudes. Thus, the effects in the current study are at least as strong as the effects found in the meta-analysis, although it must be acknowledged that the present study is not strictly comparable because it lacked a control group.

This study had a number of limitations that must be acknowledged. As well as the lack of a control group, participants were self-selected community members who enrolled themselves either by completing an expression of interest form or contacting the trainer and ANPL committee members. The evaluation also did not include a follow up to see if any changes were maintained beyond the post-training evaluation and whether there were any improvements in helping behaviour.

Despite these limitations, the findings from the current study are consistent with studies undertaken with other multicultural communities, as well as mainstream Australian and other Western cultural groups [2];[9];[10];[19];[20]; [23]; [24]; [25]. While this is the first study carried out in Australia to measure the impact of MHFA in the Nepalese community, similar studies have been undertaken in Chinese and Vietnamese communities in Australia and with the Bhutanese refugee community in the USA.

The evaluation of MHFA with the Vietnamese community [25] and the Chinese community [20]; [24] in Australia found that the training produced improvement in knowledge about mental illness including recognition of mental disorders, reduced negative attitudes to person with mental illness and improved knowledge about early intervention to a person experiencing mental illness.

Our findings are consistent with the previous evaluations conducted in Australia [20]; [24]; [25] and the USA [10]; [9] as well as the findings of a systematic review and meta-analysis of MHFA training, which also found that MHFA training led to improvements in MHFA knowledge, recognition of mental illnesses, and beliefs about effective treatment, and a small reduction in stigmatizing attitudes [2].

These studies had varying approaches to implementation, ranging from using a native speaker as the MHFA instructor to using an interpreter, and from translated resources in the target group language to resources in English but delivery in native language. Despite this variation, all the studies found MHFA to be effective in at least two aspects of mental health literacy.

The findings of the evaluation of MHFA training in the Bhutanese refugee community in the USA [9] were similar to findings of the evaluation conducted- with the Chinese and Vietnamese communities in Australia, except in the case of reduction of stigmatizing attitude towards a person with mental illness. The authors suggested that this could be due to issues with the training delivery. Subsequent changes were made, such as delivery of the training by a native MHFA Instructor and cultural adaption, which produced as significant reduction in stigmatizing attitude [10]. The cultural adaptation training was organized for prospective MHFA participants from the resettled Bhutanese refugee community in the USA and included explanation of commonly used Nepali words to describe mental health issues, and videos of community members from the Bhutanese community who had lived experience of mental health issues. These videos explained that they got help and are on a recovery journey, explained what helped them and how best to speak with someone who is experiencing psychological difficulties. A similar approach, especially on common language and expression by Nepalese community members, was used as part of the Standard MHFA training with the Nepalese community in Australia. The Standard MHFA course is designed for 12 h over two days, however with the Nepalese community, the training generally lasted for about 14 h due to the need to explain in the Nepali language and provide examples of appropriate words to describe particular mental health issues in Nepali language.

Participants found the course content to be highly relevant (4.82 out of 5), could easily ask questions to the instructor (4.91 out of 5) and rated the instructor’s knowledge highly (4.93 out of 5). However, information in the program was rated less highly (4.01 out of 5) compared to the other three areas. This may indicate that the participants may have had prior knowledge, education or professional exposure in the area of mental health. The participants included 15 registered nurses or nursing students and 30 social workers and social work students, and 20 respondents stated that they had attended mental health training prior to attending MHFA training, which may have affected these rating or contributed to ceiling effects on some of the other outcomes. Future programs should focus on those who may not otherwise attend this training, such as non-health professionals from the Nepalese community.

Despite the significant improvements in knowledge related to mental health issues, confidence and intention to extend help to the people dealing with mental illness, we did not assess whether this intention to help was converted to actual helping behavior, and this needs to be further investigated. Furthermore, the deep-rooted stigmatizing attitudes about mental illness require much more effort. MHFA Australia may need to consider some adaptation of the course for multicultural communities where there are strong stigmatizing attitudes towards people with mental illness.

A previous study conducted in the UK also showed that stigma and cultural taboos are barriers to people disclosing mental health issues and prohibit further discussion of this topic among the healthier ones [8]. This is consistent with a study showing a lower level of change in stigma among Bhutanese refugees, who share similar cultural aspects to the Nepalese [9]. A previous evaluation study of MHFA delivered by non-native instructor for resettled Bhutanese refugees in the USA found that there was no improvement in the stigmatizing attitudes among training participants [10].

## Conclusion

MHFA training in the Nepalese community in Australia has demonstrated large improvements in knowledge, confidence and intention, to help. There were also medium improvements in social distance and stigma items about signs of weakness, and small improvements in recognition of depression and the stigma items on “not a real illness”, “dangerous” and “would not tell anyone”. The overall finding is that MHFA training is effective on many levels, but that there are some areas for improvement.

Future implementation needs to consider the implications of these findings for improving the training. The weaker effect on recognition of depression could possibly be linked with a lack of native vocabulary about different mental health issues, as often Nepalese community members use the terms ‘stress’, ‘anxiety’ and ‘depression’ interchangeably. Similarly, the effect on stigma reduction needs to be considered in the future implementation of MHFA in the Nepalese community, including the perception of people with mental illness as being dangerous. In order to improve overall effectiveness in the Nepalese community, cultural adaptation on stigma reduction and focus on recognition of depression and other common mental illness may need to be considered as part of future MHFA training for Nepalese community participants. The addition could cover broader socio-cultural, traditional and religious belief issues rather than MHFA alone.

The accumulated evidence base of MHFA training for general community members should be broadened for a wider community. Improved ability to recognise mental illness, and basic skills about rendering assistance to persons experiencing mental illness, would enhance first aid coverage in the community for mental health issues and help early intervention and help seeking in newly established and emerging communities in Australia.

## Footnotes

1 https://mhfa.com.au/courses/public/types/standardedition4.
